# Redefining Professionalism in Disability Services: Digital Transformation, Boundary Work, and Professional Capital in Frontline Care During the COVID‐19 Pandemic

**DOI:** 10.1111/jar.70196

**Published:** 2026-02-15

**Authors:** Richard Gäddman Johansson, Kristina Engwall

**Affiliations:** ^1^ Department of Social Work Stockholm University Stockholm Sweden; ^2^ Department of Social Work Uppsala University Uppsala Sweden

**Keywords:** boundary work, COVID‐19 pandemic, day centre services, digital technology, professional capital

## Abstract

**Background:**

During the COVID‐19 pandemic, frontline care workers in day centre services for adults with intellectual disabilities rapidly adapted their roles through use of digital technologies. This study examines how these developments shaped professional roles and identities.

**Method:**

The study draws on qualitative interviews with 14 frontline staff and 5 managers in day centres that shifted from on‐site to remote services. Thematic analysis was guided by the concepts of boundary work and professional capital.

**Results:**

Digitalisation temporarily reshaped professional identity by enabling increased digital competence, peer collaboration, and creative agency. Staff experienced tensions between digitally mediated practices and values tied to co‐presence, embodied interaction, and relational care.

**Conclusions:**

Digitalisation created both opportunities and strains in frontline care work. Sustaining constructive digital practices will require organisational support that recognises frontline workers' expertise, integrates digital competence with reflective professional development, and involves staff and service users in shaping how technologies are used.

## Lay Summary

- During the COVID‐19 pandemic, frontline care staff working in day centre services for adults with intellectual disabilities rapidly shifted to digital ways of working, which temporarily changed how they carried out and understood their professional roles.
- Digital technology enabled staff to maintain contact with service users, collaborate with colleagues, and experiment with new forms of support.
- At the same time, staff experienced tensions between remote, technology‐mediated practices and core caregiving values such as co‐presence, embodied interaction, and relational attentiveness.
- To make constructive use of digital technologies in day centre services, organisations need to provide ongoing support that strengthens staff's digital and reflective skills and ensures that both staff and service users can influence how digital technologies are introduced and used.

## 1 Introduction

The rapid spread of the COVID‐19 pandemic in the spring of 2020 profoundly affected the provision of social care and services targeting people with intellectual disabilities (Chen et al. 2022; Linehan et al. 2022; McMahon et al. 2020). In Sweden, the government adopted a voluntaristic pandemic strategy, relying on recommendations rather than mandates (Bylund and Packard 2021; Ludvigsson 2020). While no national suspension of disability services was formally imposed, people with intellectual disabilities were designated by the National Board of Health and Welfare (NBHW) as a particularly vulnerable group (NBHW 2021). As a result, many municipal authorities across the country implemented local restrictions to disability services. For instance, about one‐third of Sweden's 290 municipalities halted or curtailed the provision of support with meaningful activities (Tideman and Aspling 2021). This change was sudden, with some frontline staff reporting no more than a day's notice (Gäddman Johansson and Engwall 2025), abruptly limiting service users' access to what is ordinarily a rights‐based service.

Care is never only a set of tasks but rather a political and ethical practice, raising questions about responsibility and distribution (Tronto 1993). The provision of support with meaningful activities is the most utilised form of service provision regulated under the Act on support and service for persons with certain disabilities (Swedish Code of Statutes 1993: 387), commonly referred to as the Disability Act, engaging approximately 42,000 service users across Sweden (NBHW 2023). Meaningful activities constitute a multifaceted form of service that is organised through day centres in Sweden. The services provided at day centres occupy a distinctive position between care and employment that offers structured, meaningful activities for adults with intellectual disabilities outside the regular labour market while also promoting social participation, personal development, and a sense of being part of a community (NBHW 2024a).

The professional role of day centre staff is demanding and contested, combining pedagogical, relational, and caregiving responsibilities, often with limited recognition (Ineland 2020; Svanelöv et al. 2019). While research conducted during the pandemic has highlighted the health risks and emotional toll experienced by frontline care workers (e.g., Chen et al. 2022), structural and potentially lasting shifts in professional roles and identities have received less attention. In Sweden, as elsewhere, frontline care workers in disability services faced staff shortages, disrupted routines, increased workloads, and redefined core responsibilities (Linehan et al. 2022). For day centre staff in particular, following the halted provision of support with meaningful activities, some became redeployed to residential care services to help cover staffing needs while others remained at day centres, exploring alternative ways for supporting service users remotely using digital technologies (Gäddman Johansson and Engwall 2025).

While much research conducted during the pandemic shows how closures and changes to remote forms of service provision had adverse effects for people with intellectual disabilities, including isolation and exclusion from everyday activities (Miettinen 2024; van Holstein et al. 2023; Vromans et al. 2023), some studies report mixed or differentiated outcomes (Chadwick et al. 2022; Gäddman Johansson and Engwall 2025). In this context, the shift from on‐site to online and digitally mediated forms of engagement reshaped the working conditions and professional practices of frontline care staff working in day centres. With many service users finding themselves in imposed lockdown within residential care services (Tideman et al. 2021), staff who remained at day centres began reimagining their roles using digital technologies such as smartphones and tablet devices to provide altered services in the form of video calls, text‐based interaction, pre‐recorded content, and live‐streamed activities (Gäddman Johansson and Engwall 2024). Digital technologies thus became essential for sustaining contact and support.

Digital technologies were already used in disability services prior to the pandemic, typically to enhance communication or promote inclusion (Isaksson and Björquist 2021; Reichenberg 2016). More broadly, health and social care services have been largely affected by digitalisation, which in services for people with intellectual disabilities raises concerns about equity and exclusion (Caton and Chapman 2016; Lussier‐Desrochers et al. 2017). Digitalisation reshapes boundaries of work and care, reconfiguring where and how tasks are carried out, creating both opportunities and new pressures (Richardson 2018). Studies of digitally mediated care highlight how technologies alter relational dynamics and professional identities, challenging boundaries between formal and informal, technical and affective labour (Crovara 2023). This aligns with scholarship on ‘care landscapes’, which emphasises how care is negotiated across shifting institutional, spatial, and cultural boundaries (cf. Carlsson et al. 2022). The pandemic thus accelerated both technology use and debates on how care, participation, and professional identity are reconfigured in an increasingly digitalised welfare context (O'Donnell et al. 2025).

This study explores how these developments—what we call a ‘digital leap’—affected the professional roles and identities of frontline care workers in Swedish day centres during the COVID‐19 pandemic. More than a temporary adjustment, the leap reflects broader societal trajectories in which digitalisation reshapes welfare services and professional boundaries. Against this backdrop, we ask:

- How did frontline care workers perceive the shift from on‐site to digitally mediated service provision?
- How did the digital transformation of service provision during the pandemic impact their professional roles and sense of identity?

The study is based on interviews with frontline staff and managers at five Swedish day centres that adopted digital technologies during the pandemic. We analyse how staff experienced this digital leap and how it shaped evolving understandings of professional identity, responsibilities, and care practices. These shifts are considered within the broader trajectories of digitalisation and professionalisation in welfare services (cf. Zander et al. 2023).

### 1.1 Frontline Care Work in Day Centres for Adults With Intellectual Disabilities

In Swedish day centres, frontline workers support service users with ‘meaningful activities’ (*meningsfull sysselsättning*) aimed at sensory stimulation, personal development, and belonging (NBHW 2024b). Activities range from communication and creative projects to gardening or vocational training, requiring a broad range of skills from staff. Some roles require more specific skills (e.g., baking or carpentry), while others require general competencies in communication and care. According to the NBHW (2024a), staff should be familiar with the values of the UN Convention on the Rights of Persons with Disabilities (United Nations 2006) and competent in health promotion, personal care, and social interaction. Crucially, this knowledge must be applied in daily interactions with service users.

Recent national efforts have sought to streamline occupational titles and define required skillsets. Yet such professionalisation has been met with ambivalence among both researchers and practitioners. Some have argued that higher education fosters ethical reflection and critical thinking (Hewitt and Larson 2007), while others have emphasised the significance of aptitude, flexibility, and situational awareness, qualities that are not easily certified (Dunér and Olin 2011; Gäddman Johansson and Hellström Muhli 2018). Studies show that some staff experience professionalisation as empowering, while others view it as constraining or misaligned with relational aspects of care (Dunér and Olin 2011; Järkestig Berggren 2015). Järkestig Berggren (2015) argues that newer roles, such as personal assistants, reflect a shift towards what she terms ‘user‐mandated professionalism’: a form of professionalism that relies on service users' knowledge as the basis for expertise rather than academic credentials or managerial standards (cf. Evetts 2010). This perspective highlights that contemporary professionalisation processes can be organised around different kinds of knowledge bases and therefore develop along multiple, sometimes competing, trajectories (cf. Brante 1988).

This ambiguity underscores broader tensions in frontline care work, which sits at the intersection of care, pedagogy, and inclusion. Staff must work to balance standardised practices with commitments to person‐centred care (Gäddman Johansson 2025), and in doing so may act as both facilitators and barriers to inclusion (Clegg and Lansdall‐Welfare 2010; Shakespeare et al. 2018). The introduction of digital technologies further complicates these tensions, as their use can empower service users but also disrupt organisational routines (Solis Lovekvist et al. 2024).

In parallel with wider debates on digitalisation, disability services are confronted with questions about digital inclusion, since people with intellectual disabilities remain at risk of exclusion (Caton and Chapman 2016; Lussier‐Desrochers et al. 2017). For frontline care staff, this sometimes means added responsibilities for supporting service users' digital access and participation, tasks that extend beyond the traditional remit of their job description and can cause tensions when training, resources, or motivation are lacking (Ramsten et al. 2017). These tensions challenge the broader integration of digital technologies in everyday disability services.

## 2 Theoretical Framework: Evolving Professional Boundaries and Capital

This study adopts a sociological lens viewing professional identities and boundaries as context‐dependent, dynamic, and relationally shaped (Blok et al. 2019; Brante 1988; Hargreaves and Fullan 2012; Liu 2018), formed through negotiation and ongoing redefinition. To understand the shifting roles of frontline care workers in disability services, particularly in response to digitalisation, two theoretical concepts are employed: boundary work and professional capital.

### 2.1 Boundary Work: Demarking and Reshaping Professional Domains

Originally from the sociology of science (Gieryn 1983), ‘boundary work’ analyses how professions define and protect their jurisdictions: areas of responsibility and expertise (Abbott 1988). Boundaries are continuously constructed and reconfigured through professional action, conflict, and collaboration.

Liu (2018) identifies three forms of boundary work: (i) ‘boundary making’, referring to conflict‐driven demarcation to assert authority; (ii) ‘boundary blurring,’ referring to collaborative blending of roles, often through hybridisation; and (iii) ‘boundary maintenance’, denoting external regulation of boundaries by institutions like the state.

In this study, boundary blurring is of particular interest. As digital technologies become embedded in care practices, traditional divisions of labour are disrupted. Frontline care workers increasingly perform tasks outside their traditional remit—so‐called ‘task shifting’—as roles evolve and new forms of care delivery emerge (Abbott 1995; Eliassen and Moholt 2023). Boundary work explains how workers navigate this flux in terms of conflict and cooperation.

### 2.2 Professional Capital: Resources for Professional Practice

To further explore how professional identities evolve, especially amid technological change, this study draws on the concept of ‘professional capital’ (Hargreaves and Fullan 2012). Originally from the context of education, this concept outlines three interrelated forms of capital: (i) ‘human capital,’ referring to individual knowledge, skills, and experience, often formalised through education and qualifications; (ii) ‘social capital,’ referring to the quality of relationships and collaborative learning; and (iii) ‘decisional capital,’ denoting the capacity for professional judgement in complex or uncertain situations.

These forms of capital shape both action and recognition in professional life. In an increasingly digitised welfare service context, professional capital extends beyond care‐related expertise to include digital skills and adaptive decision‐making in technologically mediated environments (cf. Solis Lovekvist et al. 2024).

When digital technologies become central to care delivery, both jurisdictional boundaries and foundational professional capacities are challenged. A combination of the concepts of boundary work and professional capital offers a framework for understanding how frontline care workers respond to these shifts. By negotiating tasks and redefining what constitutes valid knowledge and competent practice in an increasingly digital care landscape.

## 3 Methods and Data

This study uses a qualitative design to explore how the digital leap during the COVID‐19 pandemic affected professional roles and identities among frontline care workers in Swedish day centres for adults with intellectual disabilities.

### 3.1 Participants and Recruitment

We conducted semi‐structured interviews (*n* = 19) with 14 frontline staff and 5 managers from Swedish day centres that adopted digital technologies during the pandemic. Five day centres were identified via professional networks and recruited through snowball sampling (cf. Biernacki and Waldorf 1981). Managers received written study information and could request a video meeting. Four centres were municipal; one was privately run under municipal contract. Three centres supported adults with severe intellectual disabilities (approximately 30 service users each) with sensory, creative, and movement‐based activities; two served adults with mild to moderate intellectual disabilities (12–15 service users) with work‐like and creative tasks. Participants varied in gender and length of employment; all but one frontline worker had been employed at their centre prior to the pandemic (see Table 1).

**TABLE 1:** Overview of information on research participants and research sites.

Participants	Frontline care staff	Day centre managers	Total	
			*n*	%
Gender				
Female	9	4	13	68
Male	5	1	6	32
Length of employment				
< 2 years	1	0	1	5
2–5 years	7	2	9	47
6–10 years	1	1	2	11
> 10 years	5	2	7	37

The types of support and remote services being provided to service users during the pandemic varied between the different day centres. However, some common activities were regular contact through voice or video calls and the creation of digital artefacts in the form of edited recordings. Some day centres also engaged with service users in real time using digital technologies, either by broadcasting musical performances online or through group activities organised through the use of digital spaces where a number of service users could interact with each other as well as the day centre staff. A key feature at several day centres was an ambition to replicate ordinary services digitally despite not meeting in person.

### 3.2 Data Collection

Data were gathered between April 2022 and February 2023, when many restrictions were relaxed and services often operated in hybrid formats. Because people with intellectual disabilities had been identified as a particularly vulnerable group (NBHW 2021; cf. World Health Organization 2020), municipal authorities instructed day centres to stagger service users' return to on‐site activities. At first, those living closest to the centres or those for whom isolation in residential care was deemed especially detrimental were invited back. For others, remote service provision continued beyond the study period for various reasons, including personal preference, fear of infection, and the influence of limited guardians or family members.

Most interviews took place in person at the day centres; a minority were conducted by phone or video at participants' request. Interviews lasted between 30 and 80 min. The semi‐structured guide covered participants' experiences of providing support during the pandemic, the shift to digital formats, collaboration within and across teams, and reflections on professional role and competence. All interviews were audio‐recorded and transcribed verbatim.

Ethical approval for the study was granted by the Swedish Ethical Review Authority (Ref. no. 2021‐06646‐01). The study was conducted in accordance with ethical guidelines and codes of conduct for social science research, including recruitment of participants, informed verbal consent, anonymisation, and the storage and publication of data (Swedish Research Council 2024). All research participants and locations have been de‐identified in the data.

### 3.3 Data Analysis

Transcripts were analysed using reflective thematic analysis (Braun and Clarke 2006, 2019). After repeated readings, initial codes were generated focusing on identity, practices, and boundary dynamics. Analysis was iterative and abductive, moving between data and theoretical concepts (boundary work; professional capital) to develop themes capturing recurring patterns and tensions.

## 4 Results

Thematic analysis identified three overarching themes: (i) Ethics, uncertainty, and professional responsiveness; (ii) Digitalisation as a driver of professional reorientation; and (iii) Contested care values and strained routines (see Table 2).

**TABLE 2:** Overview of themes and subthemes.

Theme	Subtheme
Ethics, uncertainty, and professional responsiveness	
Digitalisation as a driver of professional reorientation	Social capital and boundary blurring through digital collaboration
	Digital competence as emerging professional capital
Contested care values and strained routines	Boundary making and organisational disconnect
	Digital dissonance and contested human capital

### 4.1 Theme 1: Ethics, Uncertainty, and Professional Responsiveness

The first pandemic wave disrupted routines and professional frameworks at day centres. With little notice of closures, staff were left without guidance and feared redeployment to residential care services within the district. Amidst this uncertainty, organisationally unanchored and physically distanced from service users, remote service delivery using digital technologies emerged as an improvised boundary work rooted in ethics and care values. Day centre staff described confusion, but also a determination to stay with familiar service users:

It felt more meaningful to keep working here, with our own service users, rather than being sent off to work with complete strangers in the district. So part of it was really about caring for our own service and our own users, which made us very motivated to do this. (Beatrice, frontline care worker)

Such choices exemplify decisional capital (Hargreaves and Fullan 2012): the use of professional discretion and situational judgement under uncertainty, guided by embodied knowledge of service users' needs and routines. Contact through calls, video greetings, or short recordings blurred the boundary between in‐person and digital presence, challenging the assumption that care required co‐presence (cf. Liu 2018). While welfare check‐ins of this kind had in some cases existed prior to the pandemic, they were typically more infrequent and only supplementary to direct in‐person care. During the closures, however, digitally mediated contact became the primary mode of sustaining relationships between staff and service users.

This reconfiguration also enabled reflection, documentation, and in some cases competence development:

What happened was that first we had a closure, there were no service users at the centres. [**…**] It was a pretty good work environment. You had time to write routines, to spend on quality assuring work, to pay invoices, or whatever it was you had to do and had to document. (Sofia, manager)

While much of this work involved core administrative tasks, staff also described having time for activities that normally fell aside, such as updating routines, consolidating knowledge, and engaging in online training modules. In this sense, the period functioned as an investment in human capital, strengthening skills and professional preparation. At the same time, maintaining contact with service users reaffirmed social capital, tending to the quality of longstanding relationships (cf. Hargreaves and Fullan 2012). In sum, frontline staff combined boundary blurring—reimagining presence digitally—with boundary making, insisting on continued relationships with known service users rather than redeployment. These decisions signalled both care and professional jurisdiction.

### 4.2 Theme 2: Digitalisation as a Driver of Professional Reorientation

Remote service provision using digital technologies challenged professional roles and disrupted care routines, prompting staff to mobilise new forms of professional capital and engage in boundary work.

#### 4.2.1 Social Capital and Boundary Blurring Through Digital Collaboration

With service users no longer physically present at the day centres as a result of the restricted services, staff from different units operated by the same service provider found opportunities to collaborate more than before. As one frontline care worker recalled, shortly after the initial closure, management instructed staff from different centres to all gather in one shared location. Being in such close proximity to other staff, and not having to dedicate as much of their scheduled working hours tending to service users' personal hygiene or accompanying them to and from different activities, meant that time was freed up to instead be spent on joint collaborative work that fostered creativity, collegiality, and recognition:

Basically, the whole centre came together to brainstorm. And staff from our two other locations were here [physically at the day centre] as well. So that brought us closer together as a staff group. Normally we hardly ever see each other. But now everyone was here, including our managers. So the cooperation definitely improved. (Patricia, frontline care worker)

This reflects boundary blurring (Liu 2018): a collaborative hybridisation where previously separate work units, roles, or identities converge. Here, the blurred boundaries concerned the previously distinct task and unit divisions between day centres, as staff who normally worked independently now collaborated across sites. While the data do not suggest a wholesale redefinition of roles, they do indicate a shift in how staff related to one another. The cooperative climate also strengthened social capital (Hargreaves and Fullan 2012) through collective learning. Yet it also revealed tension with the Disability Act's emphasis on individualised support:

In some frontline teams, it turned into more of a group activity that was offered [when using digital technology]. But the whole idea behind the Disability Act is individual support. […] In some ways you need to approach this with a lot of humility. It was an extremely difficult task [for the staff] to solve. (Rosa, manager)

This illustrates a tension specific to the Swedish context. According to the Disability Act, services are to be person‐centred and tailored to the individual, making collective formats the exception rather than the norm. Group‐based digital activities, though pragmatic, departed from this principle. Staff stressed such formats could not replace daily encounters with service users, underscoring that gains in collaboration facilitated by digital technologies came with trade‐offs.

#### 4.2.2 Digital Competence as Emerging Professional Capital

As digital tools became central, skills like online communication, filming, and editing gained new professional value. Staff described learning new digital routines collectively, experimenting together, troubleshooting in groups, and supporting one another as they adapted to using new tools. Digital skills emerged as forms of human capital, with staff describing instances of learning from one another:

In the beginning, we sat in different rooms with headphones on, being like “Can you hear me? Do you see me?”, “No, you can't see it there”, and “How come you can't see?” I think, generally speaking, digital skills in the workplace have improved quite a lot. From people being afraid to even turn on a computer or document something, because it can feel difficult and scary, to being able to invite others to a [digital] meeting and lead an activity. (Patricia, frontline care worker)

In this sense, human capital was expanded collectively. For some staff, digital work reshaped self‐understanding:

Personally, as a staff member here, I now feel I have more to offer [after having worked both in front of and behind the camera]. I've let go of my own sense of prestige. I'm not uncomfortable doing things like that anymore. […] I think it makes me, and us as a frontline team, more open and better [at our job]. (Andreas, frontline care worker)

This reflects decisional capital, but not in the sense of formal decision‐making authority. Rather, staff were required to exercise everyday professional judgement in situations where routines were absent or incomplete. As digital service provision became the only option, staff needed to decide how to structure online activities, troubleshoot in real time, and determine what constituted adequate support. Andreas' increased confidence in working both in front of and behind the camera illustrates how such everyday decisions became easier as digital competence grew. Staff also used filming to reflect on practice, strengthening both technical competence and professional insight:

To film something, like an interaction, and then watch it together can be a really good pedagogical tool. Seeing yourself like that becomes a kind of reflection. (Beatrice, frontline care worker)

Filming interactions provided a tool for reflective practice, enabling staff to identify what worked well and adjust their approach. This process further strengthened their decisional capital by enhancing confidence and professional judgement in shaping future care activities. Overall, digitalisation prompted not only technical upskilling but also confidence, creativity, and new dispositions. These benefits were not inherent in the technologies themselves, but emerged through situated experimentation and peer support.

#### 4.2.3 Summary of Theme 2

Digitalisation thus sustained services while fostering reprofessionalisation. Through social, human, and decisional capital, frontline care workers expanded their roles into a hybridised professionalism: grounded in care values yet reshaped by digital practices. Staff valued these developments but remained uncertain about how digital competence should be balanced with relational care, a tension explored in the following theme.

### 4.3 Theme 3: Contested Care Values and Strained Routines

While digitalisation enabled renewal and strengthened collegiality, it also exposed tensions and role strain. This theme highlights how digital formats disrupted care routines, producing contested responsibilities, questioned competencies, and unresolved ethical dilemmas. These frictions revealed broader struggles over how care should be organised, delivered, and by whom.

#### 4.3.1 Boundary Making and Organisational Disconnect

Despite their close relational knowledge of service users, frontline staff were excluded from key decisions during the initial pandemic response. Municipal directives to halt the provision of on‐site day centre services and instead shift to remote services arrived suddenly, without dialogue and with scarce information to go on:

We had no idea what was going on. In the middle of all the confusion came the sudden announcement that we were to shut down our on‐site services. And it became really, really strange. We were supposed to continue providing day centre services, but not on site. Those were the only directives that we were given. (Rosa, manager)

These top‐down orders illustrate boundary maintenance (Liu 2018), where higher organisational levels sidelined frontline staffs' experiential expertise. Frontline staff concerns about declining mental health and isolation among service users were often ignored by managers and other higher‐level decision‐makers within disability services, who had the authority to enact changes. That is, until medical staff intervened and confirmed the need for service users perceived as most at risk to receive on‐site services:

We were in regular contact with the MRN [medically responsible nurse (*medicinskt ansvarig sjuksköterska*)], who eventually came to the conclusion that mental ill‐health [among service users] was the greater concern in all of this, and decided that it was better for service users to return [to the day centre]. (Doris, frontline care worker)

Tensions also arose with residential care staff, who were reluctant to support remote participation in day centre services (e.g., setting up and operating devices). As one manager noted: ‘We've just kept at it. Pushing and pushing and pushing. Talking about what could be done and how to do it’ (Peter, manager). Although the municipal authorities instructed day centre staff to continue providing services off‐site, this directive did not translate into an operational mandate. Staff were responsible for sustaining digital participation, yet lacked the authority, resources, and cross‐services coordination needed to ensure that residential care settings supported the work. In practice, day centre staff responsibility expanded while their formal influence remained limited, increasing strain.

Accounts of this kind, depicting tensions between day centre and residential care staff, illustrate boundary making in action (Liu 2018). Here, staff in the two contexts negotiated and sometimes clashed over the limits of their respective domains: day centre staff relied on residential care staff to facilitate remote participation, while the latter were often hesitant to engage. This misalignment created fragmented services, where digital service provision depended on individual initiative and goodwill rather than organisationally supported cooperation. In such contexts, the decisional capital of day centre staff was undermined not because they lacked judgement or skill, but because they were structurally disempowered.

#### 4.3.2 Digital Dissonance and Contested Human Capital

Even when accepted as necessary, digitalisation was often felt by day centre staff as misaligned with relational care. Some described emotional loss:

You still want that personal element somehow. To have a real person behind the other screen. Especially when you don't know who is actually there, when you're talking and so on. It's something completely different when you meet them in person. (Jenny, frontline care worker)

Others voiced principled resistance, a sense that digital formats diluted the authenticity and spontaneity that define good care:

I have a bit of resistance towards these devices. But I've had to learn. And it's not that hard if you really have to do it. […] But physical work is more enjoyable than digital work. That's just how it is. (Karin, frontline care worker)

Frontline staff worried that core care capacities—attentiveness, presence, emotional sensing—might be devalued if digital competence became the main marker of professionalism. Practical concerns reinforced this view:

You have to be a bit attentive and responsive, think about when the last time was that I had a proper conversation with this person. […] And then maybe something major has happened that needs to come out. (Greta, frontline care worker)

Managers also acknowledged competence gaps: ‘We need to take a broader approach to technology and digitalisation. And above all, to assistive products for our participants’ (Ulla, manager). Thus, many defended embodied interaction not to reject digital tools, but to safeguard the ethical and affective core of care against what they perceived as an emerging *digital‐first* logic: an organisational prioritisation of technical solutions as the self‐evident way forward.

#### 4.3.3 Summary of Theme 3

The digital transition, while generative for some, also created strain and contested values. What felt like innovation in one setting was experienced as erosion in another. Professional capital was reconstituted, but also questioned. Staff mobilised decisional and social capital to sustain care digitally, while also negotiating the growing emphasis on digital competence in their professional roles. Their ambivalence reflects a dual concern: embracing digital technology pragmatically, yet remaining attentive to the relational and care‐focused aspects central to their work.

## 5 Discussion

The findings raise a central question for disability services after the COVID‐19 pandemic: what kind of professional identity and care ethos is emerging in an increasingly digitalised welfare context? This relates to wider trajectories of welfare digitalisation, where disability services are drawn into debates on digital inequalities and exclusion (Caton and Chapman 2016; Lussier‐Desrochers et al. 2017). The pandemic accelerated not only a temporary work reorganisation but also broader societal shifts. Analytically, this can be interpreted alongside international research on how digitalisation redraws work boundaries, blurring distinctions between professional and personal, formal and informal, or technical and affective labour (Crovara 2023; Richardson 2018). It also highlights what Tronto (1993) calls the politics of care: struggles over responsibility distribution and valuation.

The study shows how frontline care workers renegotiated professional roles under crisis, developing new competence, expanding agency, and engaging in cross‐boundary collaboration. Digitalisation acted as a catalyst, prompting temporary forms of reprofessionalisation (Embregts et al. 2021; Thalen et al. 2023), though unevenly. Restructuring professional capital generated ethical tensions and contested understandings of good care, producing a layered transformation in which professional values were both reimagined and resisted.

For some staff, the pandemic created opportunities for creativity, leadership, and peer collaboration (Embregts et al. 2021), challenging notions of professionalism tied to credentialed expertise (Davies 1995). The digital leap foregrounded forms of situational judgement, adaptability, and user‐oriented knowledge, aligning with alternative professionalisation trajectories such as ‘user‐mandated professionalism’ (Järkestig Berggren 2015). Digital competence became a form of professional capital (cf. Hargreaves and Fullan 2012), echoing how crises often trigger occupational reinvention (cf. Liu 2018; O'Donnell et al. 2025). Boundaries between sectors, professions, and spaces of care had to be renegotiated (cf. Carlsson et al. 2022).

Yet some feared that emphasis on digital skills risked sidelining core aspects of care, such as co‐presence, empathy, and embodied attunement (cf. Dunér and Olin 2011; Söderström et al. 2023). Staff continued prioritising relational care during the shift to remote services but questioned whether these values would remain recognised in an increasingly digital context. The digital leap altered the conditions for enacting care, prompting staff to reassert its meaning in technologically mediated forms (Bakkum et al. 2022; Scheffers et al. 2021).

This ambivalence underscores deeper questions about how care is valued and structured. Staff grappled with sustaining relational, sensory, and situational elements of care digitally, while managing blurred responsibilities in cross‐service work. Digitalisation unsettled assumptions about professional care traditionally tied to institutional logics, training, and bounded duties (Davies 1995). These findings align with international debates on digitalisation's impact on work boundaries (cf. Crovara 2023; Richardson 2018) and echo care ethics perspectives highlighting contested recognition and responsibility (Tronto 1993).

Research has noted potential benefits for people with intellectual disabilities arising from digital leaps during the pandemic (Chadwick et al. 2022; Seale 2023). As many services have reverted to on‐site provision, questions emerge about how newly acquired digital competences can be sustained in the post‐pandemic era (O'Donnell et al. 2025; Seale 2023). Similar questions apply to frontline care workers regarding professional boundaries and capital. The present study does not assess the persistence of these shifts but highlights the forms of professional change visible during the crisis.

Looking forward, structural support for sustaining newly acquired digital and professional capacities is essential. Such support includes organisational routines, reflective spaces, and prioritisation that allows digital competences to be maintained and used in everyday practice. Hybridised professionalism entails more than credentialed digital expertise; it requires blending competencies, values, and voices, with ongoing opportunities to utilise skills in service provision to the benefit of service users. Service providers should enable frontline care staff to reflect on how digitalisation shapes the content and outcomes of their work and relationships with service users.

Digitalisation of disability services represents both opportunity and responsibility. It allows for reimagining and expanding frontline care workers' professional identity while demanding that people with intellectual disabilities have meaningful opportunities to engage with and influence digital technologies, counteracting digital exclusion and persisting inequalities (cf. Caton and Chapman 2016; Gäddman Johansson and Engwall 2024; Lussier‐Desrochers et al. 2017).

### 5.1 Limitations and Future Research

This study offers valuable insights but has limitations. It focused on day centre services that had already implemented and, to varying degrees, embraced digital technologies. Future research should also include services that resisted or did not implement digitalisation during the pandemic. Comparing digital and non‐digital practices would provide a fuller picture of how they evolve, coexist, or diverge over time, and how sustainable and ethical digital care can be developed.

## 6 Conclusion

This study shows how the COVID‐19 pandemic temporarily reshaped day centre services for adults with intellectual disabilities in Sweden, bringing new forms of work, collaboration, and professional role negotiation to the fore. The digital leap reconfigured everyday routines and challenged taken‐for‐granted assumptions about co‐presence, competence, and what counts as professional care.

On the one hand, digital practices created space for creativity, peer collaboration, and new forms of professional capital. When supported by curiosity, experimentation, and informal learning, frontline care staff developed confidence and agency in using digital tools, illustrating how crisis situations can prompt forms of professional renewal (cf. O'Donnell et al. 2025; Thalen et al. 2023).

On the other hand, the shift also revealed unresolved challenges: limited involvement of frontline workers in organisational decisions, uncertainties about professional identity, and risks of unequal access for service users with limited digital skills or support (Vromans et al. 2023). As previous research has shown, the ethics of care become strained when technology complicates rather than facilitates participation (Bakkum et al. 2022; Solis Lovekvist et al. 2024).

Taken together, the findings point to three areas of attention for future practice. First, organisations need to support dialogue around digitalisation, acknowledging frontline care workers' situated expertise and its relevance for shaping care practices. Second, professional development should integrate digital skills with reflective and relational dimensions of care, ensuring that technology complements rather than displaces core care values. Third, disability services should create practical opportunities for both staff and service users to influence how digital tools are introduced and used in everyday activities. Such involvement can help ensure that digitalisation aligns with person‐centred and rights‐based principles.

In conclusion, while this study does not assess long‐term organisational change, it highlights how the COVID‐19 pandemic made visible new tensions and possibilities in the digitalisation of disability services. The digital leap underscored the resilience and adaptability of frontline care workers, as well as their emerging role in supporting digital participation. Sustaining this role beyond the pandemic will require organisational commitment to structures that uphold dignity, participation, and equity in an increasingly digital care landscape.

## Funding

This work was supported by the Swedish Research Council for Health, Working Life and Welfare, 2021‐01880.

## Ethics Statement

The study was conducted in accordance with the ethical guidelines and codes of conduct for social science research, including recruitment of participants, informed verbal consent, anonymisation, and the storage and publication of data (Swedish Research Council, 2024). All research participants have been de‐identified. Ethical approval was granted by the Swedish Ethical Review Authority (Ref. no. 2021–06646‐01).

## Conflicts of Interest

The authors declare no conflicts of interest.

## Data Availability

The data that support the findings of this study are available on request from the corresponding author. The data are not publicly available due to privacy or ethical restrictions.
